# Assessment of Pharmacological Potential of Novel Exopolysaccharide Isolated from Marine *Kocuria* sp. Strain AG5: Broad-Spectrum Biological Investigations

**DOI:** 10.3390/life12091387

**Published:** 2022-09-06

**Authors:** Samar Zuhair Alshawwa, Khalid S. Alshallash, Ahmed Ghareeb, Ahmed M. Elazzazy, Mohamed Sharaf, Afaf Alharthi, Fathy Elsayed Abdelgawad, Dalia El-Hossary, Mariusz Jaremko, Abdul-Hamid Emwas, Yosra A. Helmy

**Affiliations:** 1 Department of Pharmaceutical Sciences, College of Pharmacy, Princess Nourah bint Abdulrahman University, P.O. Box 84428, Riyadh 11671, Saudi Arabia; 2 College of Science and Humanities—Huraymila, Imam Mohammed Bin Saud Islamic University (IMSIU), Riyadh Province, Riyadh 11432, Saudi Arabia; 3 Botany and Microbiology Department, Faculty of Science, Cairo University, Giza 12613, Egypt; 4 National Research Centre, Department of Chemistry of Natural and Microbial Products, Division of Pharmaceutical and Drug Industries, Cairo 12622, Egypt; 5 Department of Biochemistry, Faculty of Agriculture, AL-Azhar University, Cairo 11751, Egypt; 6 Department of Clinical Laboratory Sciences, College of Applied Medical Sciences, Taif University, Taif 21944, Saudi Arabia; 7 Medical Biochemistry Department, Faculty of Medicine, Al-Azhar University, Cairo 11651, Egypt; 8 Chemistry Department, Faculty of Science, Islamic University of Madinah, Madinah 42351, Saudi Arabia; 9 Medical Microbiology and Immunology Department, Faculty of Medicine, Zagazig University, Zagazig 44519, Egypt; 10 Smart-Health Initiative and Red Sea Research Center, Division of Biological and Environmental Sciences and Engineering, King Abdullah University of Science and Technology, P.O. Box 4700, Thuwal 23955-6900, Saudi Arabia; 11 Core Labs, King Abdullah University of Science and Technology, Thuwal 23955-6900, Saudi Arabia; 12 Department of Animal Hygiene, Zoonoses and Animal Ethology, Faculty of Veterinary Medicine, Suez Canal University, Ismailia 41522, Egypt; 13 Department of Veterinary Science, College of Agriculture, Food, and Environment, University of Kentucky, Lexington, KY 40503, USA

**Keywords:** *Kocuria* sp., marine drugs, secondary metabolites, natural products, exopolysaccharide, FTIR, acetylcholine esterase activity, 5-LOX, COX-2, cytotoxicity, antioxidant

## Abstract

With more than 17 clinically approved Drugs and over 20 prodrugs under clinical investigations, marine bacteria are believed to have a potential supply of innovative therapeutic bioactive compounds. In the current study, *Kocuria* sp. strain AG5 isolated from the Red Sea was identified and characterized by biochemical and physiological analysis, and examination of a phylogenetic 16S rRNA sequences. Innovative exopolysaccharide (EPS) was separated from the AG5 isolate as a major fraction of EPS (EPSR5, 6.84 g/L^−1^). The analysis of EPSR5 revealed that EPSR5 has a molecular weight (Mw) of 4.9 × 10^4^ g/mol and number average molecular weight (Mn) of 5.4 × 10^4^ g/mol and contains sulfate (25.6%) and uronic acid (21.77%). Analysis of the monosaccharide composition indicated that the EPSR5 fraction composes of glucose, galacturonic acid, arabinose, and xylose in a molar ratio of 2.0:0.5:0.25:1.0, respectively. Assessment of the pharmacological potency of EPSR5 was explored by examining its cytotoxicity, anti-inflammatory, antioxidant, and anti-acetylcholine esterase influences. The antioxidant effect of EPSR5 was dose- and time-dependently increased and the maximum antioxidant activity (98%) was observed at 2000 µg/mL after 120 min. Further, EPSR5 displayed a significant repressive effect regarding the proliferation of HepG-2, A-549, HCT-116, MCF7, HEP2, and PC3 cells with IC_50_ 453.46 ± 21.8 µg/mL, 873.74 ± 15.4 µg/mL, 788.2 ± 32.6 µg/mL, 1691 ± 44.2 µg/mL, 913.1 ± 38.8 µg/mL, and 876.4 ± 39.8 µg/mL, respectively. Evaluation of the inhibitory activity of the anti-inflammatory activity of EPSR5 indicated that EPSR5 has a significant inhibitory activity toward lipoxygenase (5-LOX) and cyclooxygenase (COX-2) activities (IC_50_ 15.39 ± 0.82 µg/mL and 28.06 ± 1.1 µg/mL, respectively). Finally, ESPR5 presented a substantial hemolysis suppressive action with an IC_50_ of 65.13 ± 0.89 µg /mL, and a considerable inhibitory activity toward acetylcholine esterase activity (IC_50_ 797.02 μg/mL). Together, this study reveals that secondary metabolites produced by *Kocuria* sp. strain AG5 marine bacteria serve as an important source of pharmacologically active compounds, and their impact on human health is expected to grow with additional global work and research.

## 1. Introduction

The Global Cancer Observatory claims that cancer has become the second leading reason for mortality globally, accounting for nearly 9.6 million deaths annually with an approximated 18.1 million new cancer cases. Cancer incidence and mortality are growing dramatically and the factors are complicated, but they reflect both population increase and ageing, while also reflecting variations in the incidence and distribution of the most prominent cancer risk factors [1]. As a result, oncology research makes significant attempts to discover new and innovative treatments that can slow the progression of cancer. Despite significant scientific and technological advances in synthetic chemistry, anticancer therapies developed from natural products continue to make significant contributions to improve the efficacy of anticancer agents and the validation of new anticancer drugs [2,3,4].

Marine ecosystems are a large and unique habitat that encompasses around 71% of the Earth’s crust [5]. Varied populations of bacteria execute vital functions for the survival of the planet’s ecosystem. Exopolysaccharide (EPS) is one of the principal organic compounds created by ocean microorganisms, which account for about half of the primary generation of organic products [5]. Isolating and identifying novel marine microbes capable of generating exopolysaccharides is becoming increasingly important. These polymers help to maintain marine environments by assisting in processes including sedimentation, particle formation, and dissolved metal and organic carbon cycling [6,7,8]. EPS play a critical role in the growth and survival of organisms in harsh environments. Apart from that, EPS are essential for nutrient uptake, aggregation, adherence to surfaces, and the production of biofilms [9]. EPS are organic macromolecules synthesized as secondary metabolites and produced externally either as slime or as a jelly-like substance. EPS may be produced as a homo- or heteropolysaccharide polymer that includes subunit configurations that may be species-specific [10,11]. EPS are mostly linear, with high molecular weights (1–3 × 10^5^ Da). The majority of known EPS are polyanionic, which mainly attributed to the presence of ketal-linked pyruvate and uronic acid, as well as inorganic residues (sulphate or phosphate) [5,12]. Additionally, the most common links between monosaccharides have been shown to be 1,4- or 1,3-linkages in stiff monosaccharide backbones and 1,2- or 1,6-linkages in the flexible ones. As well, positioning of monosaccharides and the assembly of only one polymer chains impact the physicochemical characteristics of polysaccharides. [13,14,15,16].

EPSs generated by microorganisms under harsh conditions have biotechnological potential including their pharmaceutical impact as antiviral and immunoregulatory, but also their distinctive gelling and thickening properties [17,18]. Marine EPSs have been also employed in various medical applications, including wound therapies and drug administration [19]. For example, *Vibrio diabolicus*, a marine bacterium, has been found in generating “Hyalurift” polysaccharides, which have characteristics comparable to hyaluronic acid and are used to restore bone integrity [7,20]. The EPS released by *Bacillus licheniformis* and *Geobacillus thermodenitrificans* has been employed for a medicinal immunomodulatory treatment. Furthermore, it has been shown that marine *Pseudomonas* sp. synthesizes sulphated polysaccharide B-1, which possesses anti-cytotoxic effect for human cancer cell lines. *Alteromonas* sp. strain 1545 produces an acidic EPS with potent rheological properties that could be used as a thickening agent. *Bacillus cereus* strain AG3 isolated from the Red Sea generates a hetero acidic EPS that exhibited anti-staphylococcal activity and showed potential for usage as an anti-inflammatory agent [21]. Cheng et al. isolated an exopolysaccharide from *Bacillus amyloliquefaciens* sp with a potent antitumor effect against gastric carcinoma cells MC-4 and SGC-7901 cells with IC_50_ of 19.7 and 26.8 µg/µL, respectively. As EPS have the ability to cause morphological changes inside the cells, it was suggested that that EPS possess antitumoral effects that could be associated to mitochondrial malfunction [22,23]. Sulfated EPS from *Anoxybacillus gonensis* YK25 revealed a significant anticancer efficacy for lung cancer cells [22]. Furthermore, sulfated EPS (levan) obtained from the *Bacillus megaterium* PFY-147 revealed substantial antioxidant and probiotic properties, which authenticate the future consequences of sulfated EPS in biomedical fields [24]. G. Liu and colleagues recently reported the anti-hepatic cancer invasion and metastatic efficacy of a new marine bacterial EPS (EPS11). EPS11 demonstrated a significant inhibitory action against hepatic cancer cell invasion, migration, and adhesion, which was linked to its ability to modify collagen I activity and suppress the expression of proteins that modulate the extracellular matrix-receptor [25]. Furthermore, EPS fraction from *Achromobacter piechaudii* NRC2 bacteria showed a potent anti-cyclooxygenase impact, in conjunction with substantial antioxidant activity [26]. *Achromobacter*, a gram-negative bacteria, produces EPS that also possesses a considerable anticancer activity [27]. Oxidative stress is a fundamental mechanism in Alzheimer’s disease, and thus medicines that limit oxidative damage may be especially effective in the treatment of Alzheimer’s disease [28]. It has been established that free radical-mediated lipid peroxidation activates cyclooxygenase (COX-2) [29]. Based on the abovementioned facts, EPS with antioxidant and anti-inflammatory properties could be utilized to reduce inflammation, that is critical in the neurotoxicity related to Alzheimer’s disease [30].

EPSs have demonstrated exceptional bioavailability, biodegradability, and mechanical strength [14]. As drug-delivery carriers, EPSs could be adjusted to provide precise medication release, lengthen the life of the medication in the body along with enhancing drug effectiveness. Kefiran–Alginate microspheres, a water-soluble glucogalactan (hetero-EPS) extracted from *Lactobacillus kefiranofaciens*, was utilized to allow the regulated discharge of a broad-spectrum antibiotic, ciprofloxacin. Based on in vitro investigations, ciprofloxacin was protected against gastrointestinal problems via kefiran–Alginate encapsulation [31]. EPSs, principally dextran, have been employed as a protective coating layer to promote medical device biocompatibility. VEGF was immobilised in a dextran covering for vascular application by Noel et al. [32]. The presence of VEGF in a vascular graft may entice endothelial cells to attach to vascular implants, thereby boosting vascular repair. Metal corrosion behaviour is still troublesome, despite its widespread usage in bone implants. According to Saveleva et al., covering a titanium-based implant with dextran can improve resistance to corrosion against mimicked bodily fluids [33].

Based on exceptional applicability of ESP and in continuous efforts to discover and explore new exopolysaccharides, this study aimed to extract and characterize a novel EPS from marine sediments originating in the Red Sea from marine *Kocuria* sp. strain AG5. Additionally, the isolated EPS (EPSR5) property was investigated in vitro then evaluated for antioxidant, anticancer, anti-inflammatory, cytotoxicity, as well as anti-acetylcholine esterase activity.

## 2. Materials and Methods

### 2.1. Bacterial Isolation and Sampling

All samples were acquired from the Red Sea water. Bacteria were separated by applying the serial dilution method to the collected samples [34]. To 750 mL of seawater, 20 g glucose, 1.0 g CaCO_3_, 0.8 g NH_4_NO_3_, 0.05 g KH_2_PO_4_, 0.6 g K_2_HPO_4_, 0.05 g MgSO_4_7H_2_O, 0.1 g MnSO_4_. 4H_2_O, and 0.1 g yeast extract were added and completed to 1 L [35].

### 2.2. Recognition of Bacterial Isolates

The bacterial isolates were chosen according to their capacity to produce a considerable amount of EPS [36]. For valid identification, phylogenic analysis was applied [37,38,39]. From the bacterial isolates, the genomic DNA was extracted on 1.2% agarose gel for PCR assay [40]. The primers of 16S rRNA utilized in PCR assay were as follows:
Forward primer:5′-TCCGTAGGTGAACTTTGCGG-3′Reverse primer:5′-TCCTCCGCTTATTGATATGC-3′

Using the BLAST tool, the discovered sequence of DNA was uploaded to the GenBank database at the National Center for Biotechnology Information for comparison purposes (https://www.ncbi.nlm.nih.gov, accessed on 22 March 2022). The phylogenetic tree was assembled by aligning sequences that were most similar to the bacterial isolate’s 16S rRNA sequences. The 16S rRNA gene sequences of bacteria were uploaded to the GenBank databases.

### 2.3. Production and Fractionation of EPS

*Kocuria* sp. strain AG5 was selected to the considerable manufacture of EPS. The manufacturing medium’s fermented liquid composition is 20 g sucrose, 2 g yeast extract, 4 g peptone, and 750 mL seawater. The medium was then diluted to 1 L to eliminate bacterial cells. After sample centrifugation at 4000 rpm/ 30 min./4 °C, 10% TCA was included, then the mixture was maintained at 4 °C overnight. Then, samples were centrifuged at 5000 rpm/20 min to eradicate bacterial cells. The pH of the resultant supernatant was then modified to 7.0 using a NaOH mix [41]. Following centrifugation, four volumes from cold ethanol were including to the supernatant to allow separation then collection of the crude exopolysaccharide. The deposit was redissolved in deionized water. Next, the sample was dialyzed for three days against deionized water. Absolute cold ethanol (1, 2, 3 and 4 L) was gradually added to the dialyzed sample to allow fractional precipitation. The UV absorption spectrum was utilized to test the existence of protein and nucleic acid residues [42].

### 2.4. Evaluation of EPSR5

EPSR5-FTIR spectra were acquired employing potassium bromide (KBr) pellets. A total of 2.0 mg of sample was added to 200 mg KBr applying the FTIR-UNIT Bruker Vector 22 Spectrophotometer, Coventry, UK), clarified by [43,44,45,46]. Uronic acid was detected utilising the technique described by Filisetti-Cozzi and Carpita. The colorimetric measurement of m-hydroxybiphenyl at 525 nm was used in this approach. To summarize, an EPSR5 sample was diluted with concentrated sulfuric acid (2 mL), and the produced mixture was boiled for twenty minutes at 100 °C. Then, it was let to cool down to room temperature, and the obtained mixture was mixed with m-hydroxydiphenyl (150 μL) and maintained for an hour at room temperature. The resultant mixture’s absorbance was recorded at 520 nm [47]. The sulphate amount of EPSR5 was established using the Dodgson and Price technique. EPSR5 was extracted on SDSPAGE (7.5% *w/v*), and the gel was then stained using methylene blue (0.5% *w/v*) and prepared with acetic acid (3% *v/v*) [48]. The amount of monosaccharide was calculated employing the procedures described by Randall et al. To thoroughly solubilize the polysaccharide architecture, EPSR5 was incubated with 2 M trifluoroacetic acid at 120 °C for 2 h. Following that, the mixture was diluted in methanol, and then the mixture was dried under decreased pressure. The remnant was added to water before being analysed on an Aminex carbohydrate HP-87C column (300 × 7.8 mm) utilizing water as the extractant and a rate of flow of 0.5 mL/min (Agilent 1100 Series System, Santa Clara, CA, USA). As standards, mannose (Man), glucose (Glc), d-glucuronic acid (GlcA), galactose (Gal), and d-galacturonic acid (GalA) were used [49]. To determine the average molecular weight (Mw), high-performance chromatography (HPLC) that had refractive index (RI) detection was utilized (Agilent 1100 Series System, Santa Clara, CA, USA). Determination of total hexose amine content was performed according to [50]; for 16 h, the samples were dissolved in 6 N HCl at 80 °C. Prior to closing the tubes, 100 mg of material was hydrolyzed at ambient temperature for 3 h. The hydrolysates were separated, the filtrates were dried at 75 °C at low pressure, and, in distilled water, the product was taken and dissolved. At this point, the duplicate products were mixed to yield compounds of 4–14 μg glucosamine per mL. Pipette aliquots of these compounds were placed onto columns of cation-exchange resin (Dowex 50, 200–400 mesh, Sigma-Aldrich Co., St. Louis, MO, USA). After rinsing the columns with water to eliminate non-absorbed residue, the amino sugars were rinsed with 2 N HCI. The amount of hexose amine was measured after the mixtures were dissolved with NaOH, and then condensation with acetylacetone was performed in a steam bath at 89–92 °C for 45 min. The pink colour obtained from condensation results of the hexose amine was recorded using a Unicam 1400 spectrophotometer ((Chandos Products (Scientific) Ltd.), New Mills, UK) at 530 nm after reaction with Ehrlich’s solution. By comparing the sample to the reference solution OD of glucosamine hydrochloride, the hexose amine concentration of the sample was confirmed to be glucosamine-equivalent.

### 2.5. Investigation of Antioxidant Effect Using DPPH Test

The technique is executed agreeing with Brand-Williams et al. [51]. Various concentrations of EPSR5 (100, 300, 500, 1000, and 1500 µg/mL) were utilized to determine the antioxidative action of EPSR5 in scavenging the DPPH free radicals. Each concentration was added with 2 mL DPPH solution and EPSR5. The mixture was then vigorously stirred, then kept in the dark. After that, at 517 nm, the absorbance was measured for each concentration at various intervals of time 30, 60, 90, and 120 min. The antioxidant capacity was measured in relation to ascorbic acid, which served as a control (Figure S3).

The percentage EPSR5 scavenging activity for DPPH was estimated from the following equation:

$$\%\;\mathrm{scavenging}=\frac{\mathrm{Control}\;\mathrm{Absorbance}-\mathrm{Sample}\;\mathrm{Absorbance}}{\mathrm{Control}\;\mathrm{Absorbance}}\;\times \;100$$

### 2.6. Investigation of Cytotoxicity on Various Human Cell Lines

To evaluate the cytotoxicity of EPSR5, different cell lines were used, including human liver cancer cell line (HepG2), adenocarcinoma human alveolar basal epithelial cells (A-459), human colon cancer cell line, human breast cancer cell line (MCF-7), Human Epithelioma-2 (Hep-2), and PC-3 cells (human prostate carcinoma cells). The IC50 for different cell lines were compared to that of cisplatin as a reference drug [52,53]. Each cell type was cultured in a 96-multi-well plate with concentration of 1 × 10^4^ in 100 µL of growing medium per well. Following 24 h, the cells were subjected to EPSR5 at several concentrations (0, 31.25, 62.5, 125, 250, 500, 1000, 2000, and 4000 μg/mL), but the control cell lines were treated with DMSO. The numbers of alive cells were determined after the plates were incubated in a humid chamber with 5% CO_2_ at 37 °C for a day [54]. The medium was eliminated, and then the cells were stained with 1% crystal violet dye that separated after 30 min of incubation, and the wells were rinsed with water. Subsequently, 30% glacial acetic acid was applied to the wells, and the plates were stirred on a Microplate reader (TECAN, Inc., Männedorf, Switzerland). Then, absorbance of the wells was measured using a microplate reader at a wavelength of 490 nm (SunRise, TECAN, Inc., Seestrasse, Männedorf, Switzerland). These trials were all carried out in duplicate [55,56,57]. The percent of cell viability was determined via the following formula

$$\%\;\mathrm{Cell}\;\mathrm{viability}=\frac{\mathrm{OD}\;\mathrm{treated}\;\mathrm{cells}}{\;\mathrm{untreated}\;\mathrm{cells}}\;\times \;100$$

where OD is the mean optical density.

### 2.7. Investigation of Anti-Inflammatory Property

#### 2.7.1. Lipoxygenase (LOX) Activity *In Vitro*

The inhibition effect of LOX enzyme was used to test EPSR5’s anti-inflammatory efficacy. The inhibition effect of EPSR5 against the 5-LOX enzyme was established by employing the procedure described by Granica et al. [58]. Ibuprofen was employed as a control sample medication in our study (Figure S4). In brief, LOX solution (1000 U/mL, pH 9) was combined with EPSR5 in various doses (0.98–125 μg/mL in DMSO) or with Ibuprofen drug (0–125 μg/mL in DMSO) as a reference drug [52] for 15 min at room temperature. Then, resultant solution was incubated with linoleic acid, and the absorption was evaluated at 234 nm using a microplate reader (BIOTEK; Winooski, VT, USA). After that, the percentage of LOX inhibitory activity was determined via the following equation:

$$\mathrm{Inhibition}\;\%=1-\frac{\mathrm{Sample}\;\mathrm{absorbance}}{\mathrm{control}\;\mathrm{absorbance}}\;\times \;100$$

A graph plotted the EPSR5 concentration and % inhibition in enzymatic effect. The concentration of substrate needed for reduction 50% of enzymatic activity (IC_50_) was determined from the graph.

#### 2.7.2. Cyclooxygenase (COX-2) Activity In Vitro

EPSR5’s anti-inflammatory efficacy was assessed by comparing its inhibition efficacy against the COX2 enzyme with various doses (0.98–125-g/mL in DMSO) to the Celecoxib drug (0–31.25 g/mL in DMSO) as a reference drug (Figure S5) [59]. The oxidation of *N*,*N*,*N*,*N*-tetramethyl-*p*-phenylenediamine (TMPD) by arachidonic acid in the presence of COX2 enzyme provided the basis for this experiment (EC 1.14.99.1). EPSR5 inhibition effect against the COX-2 enzyme was quantified employing the technique described by Amessis-Ouchemoukh et al. and Petrovic and Murray [60,61]. The inhibiting activity was calculated by means of a microplate reader (BIOTEK; Santa Clara, CA, USA) with the absorbance at 611 nm. Celecoxib was employed as a control medication to assess COX2 enzyme reactions. The COX2 inhibitory activity percentage was calculated as follows:

$$\mathrm{Inhibition}\;\%=1-\frac{\mathrm{Sample}\;\mathrm{absorbance}}{\mathrm{control}\;\mathrm{absorbance}}\;\times \;100$$

IC_50_ for the assay is defined as the amount of sample needed to reduce 50 % of enzymatic action.

### 2.8. Membrane Stabilization

The hemolysis inhibiting effect of ESPR5 was assessed using the approach described by Shinde et al. [62] that was dependent on RBC hemolysis generated by hypotonic solution. To create RBC suspension, blood was collected from rats employing anticoagulated syringes, rinsed with 10 mM sodium phosphate buffer (154 mM NaCl, pH 7.4), then centrifuged for 10 min at 3000× *g*. The RBCs suspension then added to 10 mM sodium phosphate buffer containing 50 mM NaCl in a volume of 0.50 mL. (5 mL, pH 7.4). The resultant solution was kept at room temperature for 10 min before being administered with EPSR5 (7.8–1000 μg/mL in DMSO) or an indomethacin drug (0–1000 μg/mL in DMSO) as a reference drug (Figure S6) [63]. Finally, the mixture was centrifuged at 3000× *g* for 10 min, then supernatant absorption was measured at 540 nm. The technique relies on assessing the capabilities of EPSR5 to reduce the percentage of reduction in hemolysis. The inhibitory percentage of hemolysis was determined utilizing the following equation:

$$\mathrm{Membrane}\;\mathrm{stabilization}\;\%=\frac{\mathrm{OD}1-\mathrm{OD}2}{\mathrm{OD}1}\;\times \;100$$

where: OD1 = Optical density of hypotonic-buffered saline solution unaccompanied, OD2 = Optical density of test sample added to the hypotonic solution.

Under the assay conditions, the amount of IC_50_ was identified as the sample concentration at which 50% of hemolysis is inhibited.

### 2.9. Acetylcholine Esterase Activity

The inhibition effect of EPSR5 towards acetyl cholinesterase activity was determined using Abcam kits (Biomedical Campus, CB2 0AX, Cambridge, UK) agreeing to the technique explained by Ellman et al. [64]. In a 1 mL cuvette, 275 μL of Tris-HCl buffer (50 mM, pH 8), 100 μL of acetylthiocholine iodide (15 mM), 500 μL of DTNB (3 mM), 100 μL of distilled H_2_O (as a blank) or reference medication Eserine (Figure S7) [52], EPSR5 with various doses (100, 250, 500, 750, and 1000 μg/mL), and 25 μL of acetyl cholinesterase reaction mixture in Tris-HCl buffer (0.28 U·mL^−1^) was put to the reaction cuvette. The absorbance of the enzymatic reaction was assessed at a wavelength of 405 nm. The following formulation was applied to calculate the inhibition % impact:

{(control absorbance − sample absorbance)/control absorbance} × 100

As a positive control, eserine was employed at the concentrations of 0.02, 0.04, 0.06, 0.08, 0.1, and 0.12 μg/mL.

### 2.10. Statistical Analysis

The information assessment was executed employing SPSS software version 22. Our records were normally distributed, as determined by the Kolmogorov–Smirnova and Shapiro–Wilk tests. Duncan’s test was employed to evaluate the similarities between various concentrations [65,66,67,68]. The data were expressed as mean ± standard deviation. *p* < 0.05 is supposedly significantly different.

## 3. Results

### 3.1. Isolation and Phylogenetic Analysis of the Bacteria

**Figure 1:**
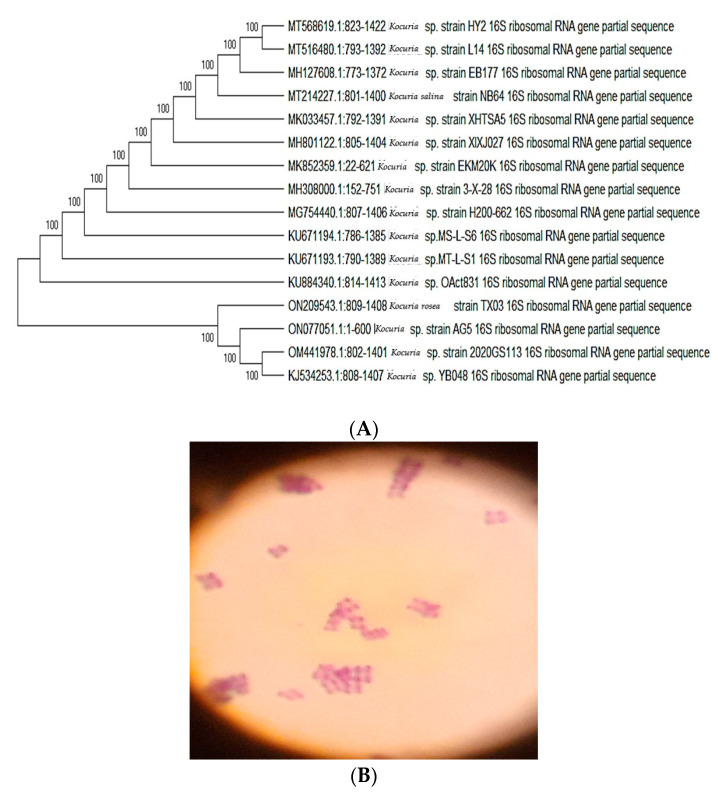
(**A**) Phylogenetic tree according to 16S rRNA gene sequencing, illustrating the phylogenetic relationship of *Kocuria* sp. within representative species of the genus Bacillus. (**B**) Gram-positive stain *Kocuria* sp. strain AG5.

In the presented work, five bacterial isolates were isolated according to the discriminant morphological features of their colonies from seawater and were developed in certain media that contains seawater, CaCo_3_, NH_4_NO_3_, MGSO_4_, MnSO_4_, KH_2_Po_4_, K_2_HPO_4_ and yeast extract [35]. Based on the conventional physiological, morphological, and biochemical investigations, the isolated strain AG5 was a gram-positive coccus, large, yellow, rough, dry, convex, short-chain, non-capsulated, non-spore-forming, non-motile, non-fast acid as well as having non-hemolytic colonies on blood agar. Three of these isolates were discovered to be capable of producing EPSs in sufficient quantities. AG5 has the greatest EPS output of the EPS yield of identified marine bacteria that considerably produced EPS (6.84 g/L). All the tests of catalase, Voges–Proskauer, Simon citrate, urease, glucose, sucrose, mannose, and Nitrate reduction showed positive yield. The recognition of isolated bacteria was established through phylogenic investigation using PCR examination using the 16S rRNA 5′-TCCGTAGGTGAACTTTGCGG-3′ primer. The phylogenetic tree was created by matching sequences with extreme resemblance to the rRNA sequences of the isolated bacterial, and the attained sequences of rRNA gene were known as *Kocuria* sp. Data of nucleotide sequences of the bacterial isolate were checked on the GenBank database. Accordingly, *Kocuria* sp. strain AG5 was identified with accession number ON077051 (Figure 1). The identified DNA sequence was submitted to the GenBank database at the National Center for Biotechnology Information for comparison purposes (https://www.ncbi.nlm.nih.gov, accessed on 22 March 2022) employing the BLAST tool.

### 3.2. Manufacture and Chemical Composition of EPSR5

The R5 bacterium separation was utilized to yield exopolysaccharide (EPS) in 6.84 g/L yield. The resultant crude residue underwent purification using fractionation and precipitation procedures. Hence, EPSR4 was treated with deionized water and the resulting solution was dialyzed for three days. Subsequently, the dialyzed solution was passed through a 100-micron ultrafiltration membrane. Absolute cold ethanol was gradually added to the dialyzed sample to allow fractional precipitation. After fractionation, the core fraction of EPSR5 (73%) was acquired in three volumes of ethanol precipitation from the crude EPS.

**Figure 2:**
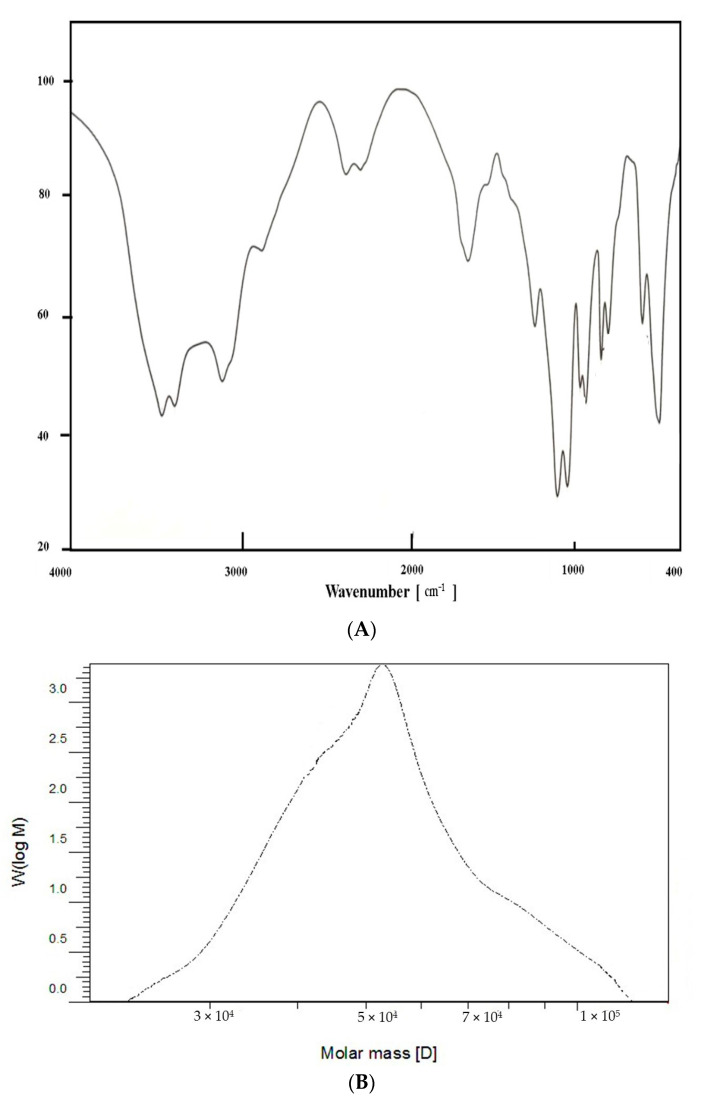
The FTIR Spectrum of EPSR5 revealing the main functional groups (**A**), and the Gel permeation chromatography analysis of EPSR5 (**B**).

The EPSR5 fraction content of sulfate and uronic acid was about 25.6 and 21.77%, respectively, while total hexose amine content was 13.55%. In addition, the fraction composition of monosaccharides included arabinose, xylose, glucose, and galacturonic acid at a molar ratio of 2.0:0.5:0.25:1.0, respectively, as revealed by HPLC. The EPSR5 fraction had an average molecular weight of 5.4 × 10^4^ g/mol and an average molecular weight of 4.9 × 10^4^ g/mol, resulting in a polydispersity index of 1.1. As outlined in Figure 2, the FTIR spectrum fraction has a broad characteristic peak at the 3447.13 cm^−1^ regions that could be linked to the expansion vibration of O–H in the sugar residue component. In addition, the fraction revealed a particular band at 1668.12 cm^−1^, assigned by circle vibrations. However, the observed band at 1129.12 cm^−1^ can be attributed to the SO = 3. Furthermore, the band at 863.953 cm^−1^ indicated the β-pyranose.

### 3.3. Antioxidant Activity of EPSR5

**Figure 3:**
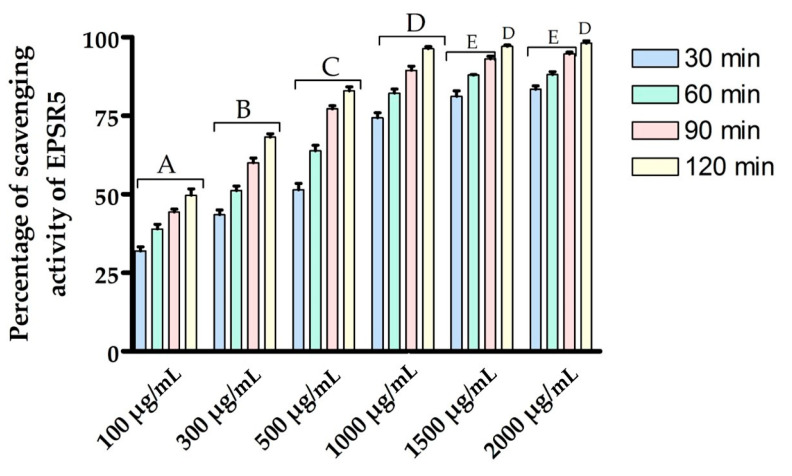
DDPH Scavenging activity of several concentrations of EPSR5 at different time intervals against (Di-Phenyl Picryl Hydrazyl) DPPH radical. Bars of the similar letters differ insignificantly (*p* > 0.05), but those that have different letters differ significantly (*p* < 0.05).

Natural polysaccharides demonstrated a considerable antioxidant activity. Toward this, DPPH experiment was performed to explore the capability of EPSR5 at various concentrations (100, 300, 500, 1000, and 1500 µg/mL) with the purpose of investigating the antioxidant activity of EPSR5. The scavenging activity of EPSR5 was measured against DPPH radicle at different time intervals (30, 60, 90, and 120 min) [51]. Accordingly, EPSR5 at various doses was subjected to a DPPH solution, and the diminishing in the absorbance of DPPH free radical was recorded utilizing a spectrophotometer at 517 nm after intervals of incubation time in the dark for 30, 60, 90, and 120 min. As demonstrated in Figure 3, the overall antioxidant effect of EPSR5 was raised in a dose- and time-dependent manner (100, 300, 500, 1000, and 1500 g/mL). At each concentration of EPSR5, a gradual elevation in antioxidant activity with increasing the time duration was observed. At each time interval, a significant peak was recorded with increasing the concentration of EPSR5. The uppermost antioxidative activity was recorded after 120 min at the concentration of 2000 µg/mL.

### 3.4. Anti-Inflammatory Activity of EPSR5

Inflammation begins as a preventive reaction in response to a variety of internal and extrinsic stressors. Chronic inflammation has been associated with a range of conditions, such as neurological problems, cancer, and cardiovascular disease. 5-LOX and COX2 are two of these regulators of arachidonic acid metabolism that have been associated to a number of inflammatory diseases [69]. The potential of 5-LOX/COX-2 enzymes to inhibit the generation of leukotrienes and prostaglandins has underlined their targeting [70]. COX-2/5-LOX inhibition works by preventing the formation of prostaglandins and leukotrienes. COX-2 inhibitors reduce inflammation by restricting arachidonic acid from being converted to prostaglandin. Arachidonate 5-lipoxygenase inhibitors block the effect of the enzyme arachidonate 5-lipoxygenase (5-LOX), which is responsible for the production of inflammatory effect.

**Figure 4:**
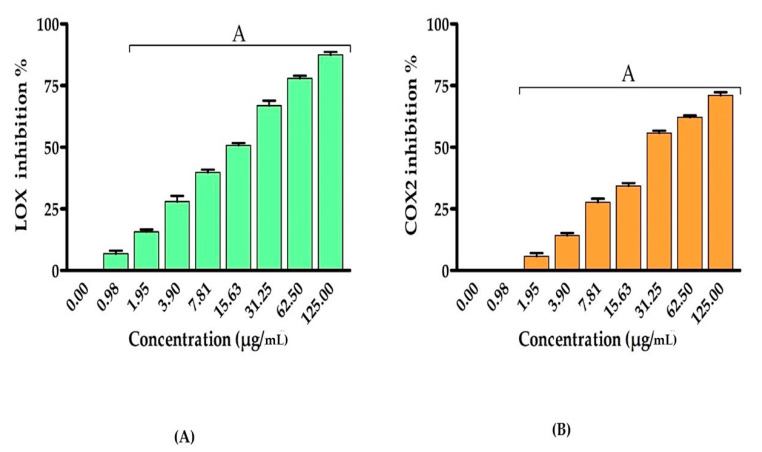
Anti-inflammatory effect of different concentrations of EPSR5 using different methods (**A**) Lipoxygenase (LOX) and (**B**) cyclooxygenase (COX-2). Each bar represents mean ± SD of triplicate measurements. Bars of the similar letters differ insignificantly (*p* > 0.05), but those that have different letters differ significantly (*p* < 0.05).

According to these findings, we wanted to explore the EPSR5 anti-inflammatory action by testing its inhibitory effectiveness against COX-2 and 5-LOX enzymes [70]. Anti-inflammatory activity of EPSR5 was estimated by assessing the changes in the activities of lipoxygenase (LOX) and cyclooxygenase (COX-2) (Figure 4). The average IC_50_ of EPSR5 on the LOX and COX-2 were 15.39 and 28.06 µg/mL, respectively. A dose-dependent elevation in the inhibition of LOX and COX-2 activities was observed by increasing the concentration of EPSR5.

### 3.5. Membrane Stabilizing Effect of EPSR5

**Figure 5:**
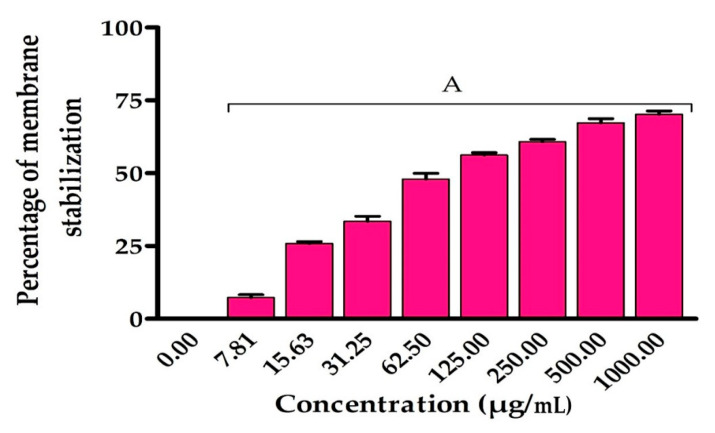
Percentage of membrane stabilization by EPSR5. Each bar represents mean ± SD of triplicate measurements. Bars of the similar letters differ insignificantly (*p* > 0.05), but those that have different letters differ significantly (*p* < 0.05).

The hemolysis suppressive action of ESPR5 was assessed using the approach described by Shinde et al., that was relied on RBC hemolysis generated by hypotonic solution [38]. In brief, the blood was drawn from rats and washed with a sodium phosphate buffer, which contains 154 mM NaCl (10 mM, pH 7.4). Subsequently, the solution was centrifuged for 10 min at 3000× *g* to afford suspension of RBCs. After that, the suspension of RBCs was added to 5 mL of sodium phosphate buffer (10 mM, 50 mM NaCl, pH 7.4). The resulting mixture was added to EPSR5 and the ability of EPSR5 to stabilize erythrocyte membranes was analysed at different concentrations (Figure 5). Interestingly, the membrane stabilization was significantly elevated by increasing the concentration of EPSR5. The LC_50_ of the percentage of stabilization of membranes was 65.13 µg/mL.

### 3.6. Antitumor Activity

**Figure 6:**
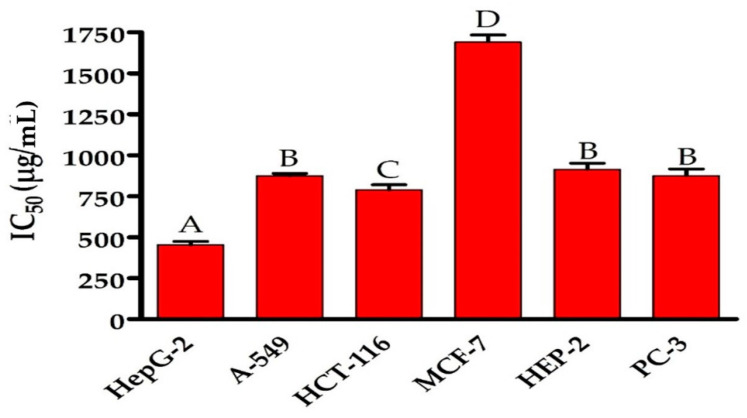
IC_50_ of EPSR5 on % viability of different cancer cell lines. Data represent mean ± SD of triplicate measurements. In each cell line, bars of the similar letters differ insignificantly (*p* > 0.05), but those that have different letters differ significantly (*p* < 0.05).

**Figure 7:**
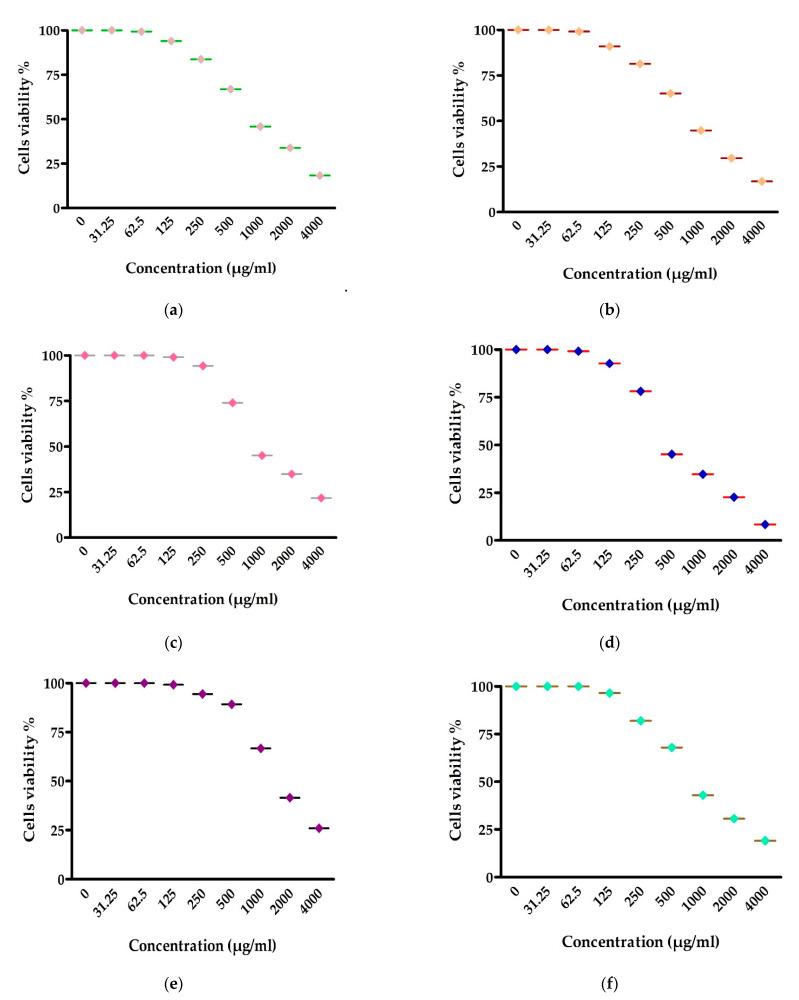
Influence of various concentrations of EPSR5 on % viability of A549 (**a**), HCT-116 (**b**), Hep-2 (**c**), HepG-2 (**d**), MCF-7 (**e**), and PC3 (**f**) cell lines. Data represent mean ± SD of triplicate measurements.

As illustrated in Figure 6 and Figure 7, the impact of various concentrations of EPSR5 on the % viability of several cell line types, including A-549 (adenocarcinoma human alveolar basal epithelial cells), HepG-2 (hepatocellular carcinoma), HCT-116 (human colon cancer cell line), MCF-7 (human breast cell line), HEP-2 (human Epithelioma-2), and PC-3 (prostate cancer cell line), were displayed according to Masamune et al.: each cell type was put in a 96-multi-well plate with a cell concentration of 1 × 10^4^ in 100 µL of growth medium per well, and the assay was executed. The highest (1691.00 ± 44.20 µg/mL) and lowest (453.46 ± 21.80 µg/mL) IC_50_ of EPSR5 for cell lines were reported in MCF-7 and HepG-2, respectively. In all the studied cell lines, gradual increase in % viability by decreasing the concentration of EPSR5. As compared to the corresponding controls, the % viability of most cell lines started to decline significantly at the level of 125 µg EPSR5/mL and continued to decline gradually by increasing the concentration. Cell viabilities were calculated according to the following formula:

$$\%\;\mathrm{Cell}\;\mathrm{viability}=\frac{\mathrm{OD}\;\mathrm{treated}\;\mathrm{cells}}{\;\mathrm{untreated}\;\mathrm{cells}}\;\times \;100$$

### 3.7. Assessment of Acetylcholine Esterase Activity

**Figure 8:**
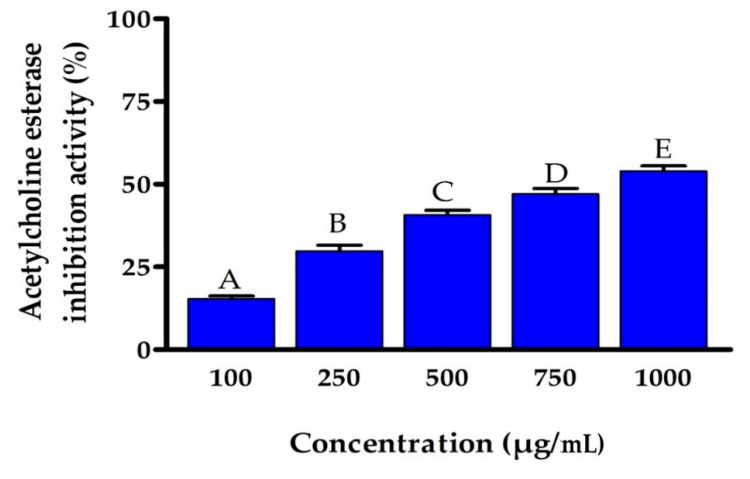
Percentage of inhibition of AChE by different concentrations of EPSR5. Bars represent mean ± SD of triplicate measurements. Bars of the similar letters differ insignificantly (*p* > 0.05), but those that have different letters differ significantly (*p* < 0.05).

The inhibition impact of the EPSR5 fraction on the AChE activity compared to eserine was evaluated as the technique stated by Ellman et al. [64] The enzymatic activity of AChE was measured at different concentrations of EPSR5. Eserine was utilized as a positive control at the concentrations of 0.02, 0.04, 0.06, 0.08, 0.1, and 0.12 μg/mL (Figure 8). The average IC_50_ of EPSR5 was 797.02 µg/mL, compared to the IC_50_ of Eserine (0.09 µg/mL). A substantial decline in the activity of AChE was recorded by raising the concentration of the EPSR5 fraction from 100 to 1000 µg/mL.

## 4. Discussion

EPS are generated by many marine bacteria as a plan for growth, sticking to solid surfaces, and surviving unfavourable environments. Separating new EPS-producing bacteria from aquatic habitats, especially severe marine environments, is becoming widely attractive due to its broad biological and pharmaceutical potential [13] as a result of our ongoing investigation of innovative bioactive compounds from marine microorganisms as well as the immunological ability of these extracts. We isolated and identified marine *Kocuria* sp. strain AG5 (Accession no. ON077051) (Figure 1) and further evaluated its EPS (EPSR5) immunological and anti-Alzheimer’s activity. Out of seven bacterial strains examined, the isolate (R5) was found to be highest producer of EPS (EPSR5). The chemical analysis of EPSR5 revealed its molecular weight to be 4.9 × 10^4^ g/mol and number average molecular weight (Mn) of 5.4 × 10^4^ g/mol comprised of arabinose, xylose, glucose, and galacturonic acid with a molar ratio of 2.0:0.5:0.25:1.0, respectively, and containing the sulfate (25.6%) and uronic (21.77%) concluding it is an acidic polysaccharide polymer (Figure 2). As mentioned above, EPSR5 has a high molecular weight of 4.9 × 10^4^ g/mol and has acidic nature. Generally, most marine EPS are linear monosaccharide polymers. The molecular weight ranges from 1 × 10^5^ Da to 3 × 10^5^ Da on average [12]. Although most EPS are neutral polymers, the bulk is polyanionic owing to the existence of uronic acids or ketal-linked pyruvate, as well as inorganic residues as phosphate or sulphate [71] as in the case of EPSR5.

Moving to EPSR5 antioxidant investigation by DDPH assay, maximum antioxidant activity was 98.11 ± 0.8% at 2000 µg/mL after 60 min (Figure 3) and reported minimum activity was 49.70 ± 2.0% at 100 µg/mL after 60 min. The higher the tested concentration, the higher the antioxidant activity yielded [72]. It is worth noting that free radical scavenging activity can also be linked to secreting enzymes such as superoxide dismutase, which aid in synthesizing glutathione, a robust non-enzymatic antioxidant [73]. Furthermore, the existence of some side chemical groups, such as sulphated group [74] and hydroxyl group [75,76], aid in the antioxidant scavenging. In agreement with our results an Eps *Bacillus albus* DM-15, isolated from the Indian Ayurvedic, the EPS displays substantial antioxidant effects on DPPH (58.17 ± 0.054%), ABTS (70.47 ± 0.854%), and nitric oxide (58.92 ± 0.744%) radicals in a concentration-dependent way [77].

Then, we discovered the anti-inflammatory effect of EPSR5 by assessing its inhibitory influence for 5-LOX and COX-2 as well as hemolysis activities. Inflammation is a preventive defence mechanism that is activated in response to numerous stressors. However, chronic inflammation has been related to a variety of disorders, including neurological disorders, cancer, and cardiovascular diseases [78]. EPSR5 anti-inflammatory test, the assay was carried out using Lipoxygenase (LOX) inhibitory test that gave IC_50_ 15.39 ± 0.82 µg/mL, cyclooxygenase (COX2) inhibitory yielded 28.06 ± 1.1 µg/mL, and finally membrane stabilization inhibitory gave 65.13 ± 0.89 µg/mL (Figure 4 and Figure 5). Microbial metabolites activate macrophages, causing them to secrete pro-inflammatory cytokines TNF-α, IL-1, and IL-6, as well as anti-inflammatory cytokine IL10, and other cytokines and their associated transcription factors [79]. For example, a lipopeptide synthesized by *Bacillus liceniformis* VS16 was found to increase IL-10 and TGF and decrease TNF-α and IL Iβ [80]. In addition, peptides isolated from *Yersinia pestis* were found to downregulate TNF-α and interleukins 12, 15 and 18 [81]. This anti-inflammatory property is thought to be due to its structure and cyclooxygenase inhibition effect [82]. It is important to note that bacteria, as anti-inflammatory inhibitors, grow faster and more naturally than those of other microorganisms on a broad basis. Furthermore, the purification of enzymes could be replaced with bacterial cell mass. [79]. In accordance with our findings, EPSR3 *Bacillus cereus* isolated from the Saudi Red Sea coast revealed anti-inflammatory activity, including the Lipoxygenase (LOX) inhibitory test which had an IC50 12.9 ± 1.3 μg/mL. In contrast, the control sample (ibuprofen) had an IC50 1.5 ± 1.3 μg/mL. Therefore, the COX-2 inhibitory test gave 29.6 ± 0.89 μg/mL, while control (Celecoxib) gave 0.28 ± 1.7 μg/mL [83]. Supporting our finding, Gangalla and colleagues have investigated the anti-inflammatory efficacy of EPS fractions (GR-1 to GR-21) generated by polluted soil bacteria. The findings revealed that EPSs fraction GR-2, GR-5, and GR-1 (65 ± 0.14, 61 ± 0.15, and 58 ± 0.38, respectively) had substantial anti-inflammatory effect when compared to indomethacin drug [84].

The MTT test was accomplished to explore the cytotoxic influence of EPSR5 against six cell lines (HepG-2, A-549, HCT-116, MCF-7, HEP-2, and PC-3 cell lines). EPSR5 showed a moderate anti-proliferative activity against breast carcinoma (MCF-7) with IC_50_ = 1691 ± 44.2 µg/mL and larynx carcinoma (HEP-2) with IC_50_ = 913.1 ± 38.8 µg/mL, whereas the considerable activity was reported in the case of hepatocellular carcinoma (HepG-2) with IC_50_ = 453.46 ± 21.8 µg/mL (Figure 6). In agreement with our findings, a recent study evaluated the cytotoxic activity of a polysaccharide EPSR5 from *B. cereus* strain AG3 against PC-3, MCF-7, and T-24 cell lines. The reported IC_50s_ were 61.4 ± 2.6 µg/mL, 55.7 ± 2.3 µg/mL, and 121 ± 4.1 µg/mL, respectively [21]. Among EPS-generating species, the most typically related EPS with promising anticancer potential were isolated from *L. helveticus, L. acidophilus*, and *L. Plantarum*. The anti-proliferative action of EPS varied from strain to strain, even within the same species [85,86]. Recently, novel EPS was isolated from *Bacillus subtilis* strain AG4 (EPSR4), which exhibited a significant anti-inflammatory activity against HepG-2, A-549, and T-24 cell lines [52]. EPS has been shown to affect or interfere with carcinogenesis-related genes such as p53, BCL2, β -catenin, and others [87]. In addition, the presence of unique structures such as uronic acid and sulphate may explain the anti-proliferative activity of EPS [88]. In our extracted polymer, EPSR5 contained sulfate (25.6%) and uronic (21.77%). Antiproliferative or cytotoxic influence is rather prevalent in sulfated EPS. Our results also substantiate with *Bacillus albus* DM-15 as an exopolysaccharide, isolated from the *Indian Ayurvedic*. The cytotoxic effect of the *Bacillus albus* DM-15 against lung carcinoma cells A549 was detected with an IC_50_ value of 20 ± 0.97 μg mL^−1^, and consequent cellular staining discovered apoptotic necrotic features in injured A549 cells [77].

Acetylcholinesterase inhibitors (AChEIs) are an appealing research topic due to their potential applications in treating neurodegenerative diseases. Fungi and bacteria were the primary sources of AChEIs [89]. Therefore, as a step forward to discover some bacterial in vitro anti-AChE activity, EPSR5 was tested at numerous concentrations (100–1000 μg/mL) with IC_50_ = 797.02 (Figure 7) compared to IC_50_ eserine control 0.09 ± 0.02. Alzheimer’s disease is the most prevalent reason for brain lesions in older people, and it causes memory and thinking skills to deteriorate over time. New research has evidenced the neuroprotective functionality of marine bacteria secondary metabolites [90]. Furthermore, experimental investigations showed that the inhibitory effect of COX-2 reduces the inflammatory process, which is significant in the neurodegeneration linked with Alzheimer’s disease progression [30]. As a result, various researchers have noted the medical use of non-steroidal COX-2 inhibitors to postpone the medical manifestation of Alzheimer’s disease. To that purpose, EPSR5’s current ability as a selective anti-cyclooxygenase, inhibitory effect against acetyl cholinesterase effect, and antioxidant capabilities may make EPSR5 a beneficial natural polysaccharide for treatment and/or restricting Alzheimer’s disease. According to our results, EPSR4 from marine Bacillus subtilis has a dose-dependent and moderate inhibitory action against acetylcholinesterase influence, with an IC50 of 786.38 g/mL when compared to Eserine with IC50 value of 0.09 μg/mL [91]. On the other hands, in Alzheimer’s disease, Acetylcholinesterase (AChE) inhibitors prevent neurodegeneration and the manufacture of reactive oxygen species (ROS) in brain cells. *Streptomyces* sp. UTMC 1334, also recognized as *Streptomyces lateritius*, was found to synthesize pyrroles and other AChE inhibitory metabolites [92]. Astrocytes shield the nervous system from the oxidative damage caused by ROS production [93]. Human primary astrocytes were found to be protected from oxidative stress by myxobacterial extracts. Indeed, pretreatment of astrocytes with myxobacterial extracts from *Archangium* sp. UTMC 4070 and *Cystobacter* sp. UTMC 4073 elevated glutathione levels, an antioxidant protein complex inside the brain [94].

Recent years have seen an enhancement in studying the potential industrial uses of EPS produced by marine microbes, although so far there have been few studies detailing their synthesis and recovery; for a complete comprehension of EPS’s qualities, more in-depth study of the topic is required. Genetic engineering (the use of mutant strains, gene alterations) may be used to enhance marine bacterial strains and hence increase EPS output, and it can also be used to manufacture EPS with desired features and structures. Gut health can be improved by bacterial exopolysaccharides by changing the makeup of gut microorganisms, boosting immunological function, and improving blood flow [95]. Additional investigations ought to be performed to verify the precise chemical structure of EPSR5 and more investigation is needed for the biocompatibility in vivo and the accurate action mechanism of EPSR5 and define whether EPSR5 can improve gut microbiome composition. Due to the rising demand for EPSs owing to abilities such as their biocompatibility, biodegradability, and non-toxicity, new EPSs are being produced by mixing them with other natural and synthetic polymers, consequently encouraging investigators to determine innovative applications in various fields.

## 5. Conclusions

In summary, our findings contribute to the knowledge on the therapeutical advantage of marine bacterial products as abundant sources of bioactive compounds, including pharmacologically active exopolysaccharides. In our current research, we have performed the isolation and characterization of a novel acidic exopolysaccharide EPSR5 derived from *Kocuria* sp. strain AG5. The isolated EPSR5 exhibited a considerable antioxidant activity as assessed by DPPH assay. Further, EPSR4 displayed a substantial inhibitory activity toward both 5-LOX and COX-2 enzymes, suggesting EPSR5 as a promising anti-inflammatory agent. Investigation of anticancer activity revealed that EPSR5 possesses a significant anti-proliferative activity against the HepG-2 cell line. Lastly, our findings revealed that EPSR5 could be a beneficial natural product against Alzheimer’s through its ability to target AChE activity. Future research should focus on analysing the exact chemical composition of EPSR5 in order to understand and elucidate the observed biological activity in relation to the metabolites produced by *Kocuria* sp. strain. These findings highlight potential feasibility of *Kocuria* sp. as well as their potential application in the pharmaceutical industry.

## Data Availability

The data presented in this study are openly available in DDBJ/EMBL/GenBank nucleotide sequence databases at https://www.ncbi.nlm.nih.gov/, accessed on 22 March 2022, reference number GenBank: ON077051.

## Supplementary Materials

The following supporting information can be downloaded at: https://www.mdpi.com/article/10.3390/life12091387/s1, Table S1. Physiological Characteristics of *Kocuria* sp. strain AG5; Table S2. Culture and Morphological Characteristics of *Kocuria* sp. strain; Figure S1: The HPLC chromatogram of EPSR5; Figure S2: The ultraviolet spectra of EPSR5; Figure S3: The antioxidant activity of ascorbic acid at different time intervals; Figure S4: The 5-LOX enzymatic activity in the presence of ibuprofen; Figure S5: The COX-2 enzymatic activity in the presence of celecoxib; Figure S6: The hemolysis activity in the presence of indomethacin; Figure S7: The acetylcholinesterase activity in the presence of Eserine.
