# Supplementary material for: Assessment of Pharmacological Potential of Novel Exopolysaccharide Isolated from Marine Kocuria sp. Strain AG5: Broad-Spectrum Biological Investigations
Source: Life (Basel). 2022 Sep 6;12(9):1387. doi: 10.3390/life12091387 (PMC9504734; doi:10.3390/life12091387)
Supplement: Supplementary file 1 [file life-12-01387-s001.zip › life-1877081-supplementary.pdf]

Supplementary Data

Assessment of Pharmacological Potential of Novel Exopolysaccharide Isolated from Marine *Kocuria* sp. Strain AG5: Broad-Spectrum Biological Investigations

**Samar Zuhair Alshawwa [1], Khalid S. Alshallash [2], Ahmed Ghareeb [3], Ahmed M. Elazzazy [4], Mohamed Sharaf [5], Afaf Alharthi [6], Fathy Elsayed Abdelgawad [7,8], Dalia El-Hossary [9], Mariusz Jaremko [10], Abdul-Hamid Emwas [11]
and Yosra A. Helmy [12,13,*]**

Table S1. Physiological Characteristics of Kocuria Sp. strain AG5.

**Physiological characteristics**	***Kocuria sp.* strain AG5**
Catalase	+
Citrate	+
Coagulase	-
Indol	-
MR(Methylred)	-
VP(Voges Proskauer)	+
Oxidase	-
Urease	+
Nitrate reduction	-
Glucose	+
Lactose	-
Sucrose	+
Mannose	+

Table S2. Culture and Morphological Characteristics of *Kocuria* sp. strain

***Kocuria sp.* strain**	Shape and Culture Characteristics
AG5	Gram positive cocci, Large, Yellow, Rough, Dry, Convex, short chains, non-capsulated, non-spore-forming, non-motile, non-acid fast and non-hemolytic colonies on blood agar

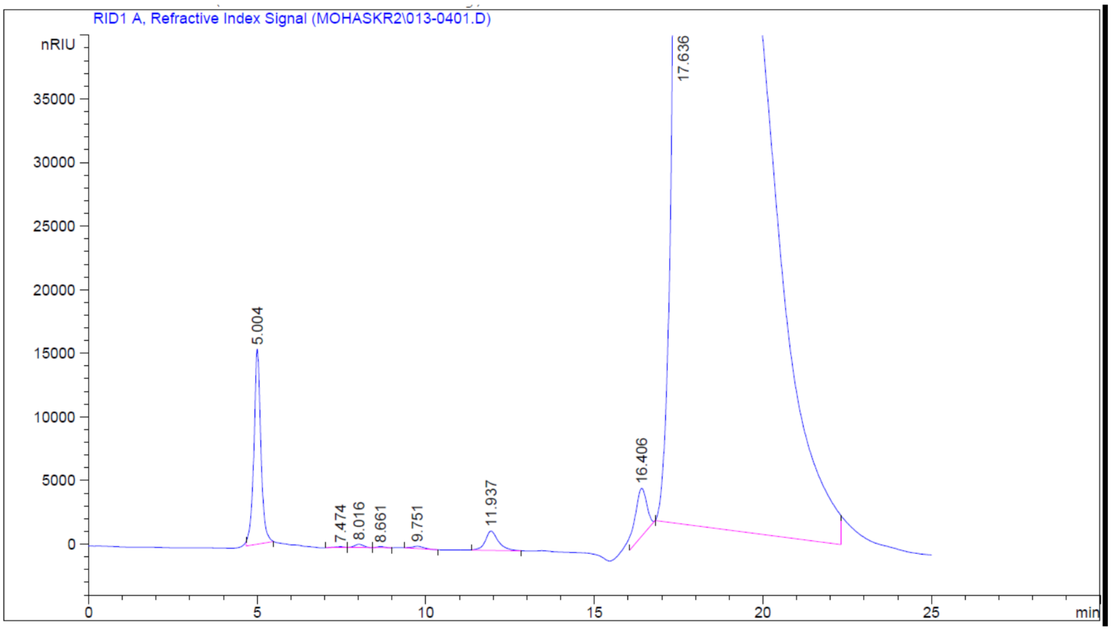

Figure S1. HPLC analysis of the EPSR5.

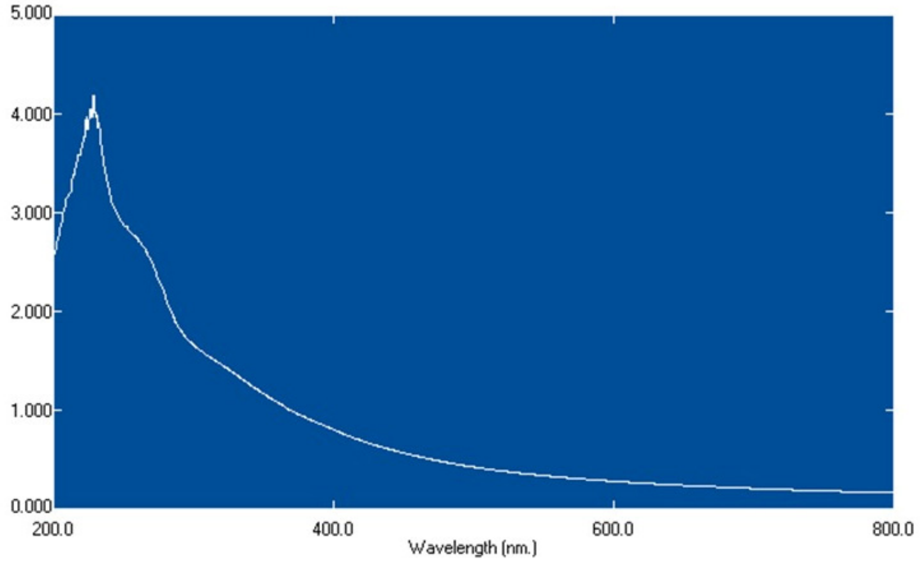

Figure S2. U.V Spectrum of EPSR5 isolated from *Kocuria Sp*. strain AG5.

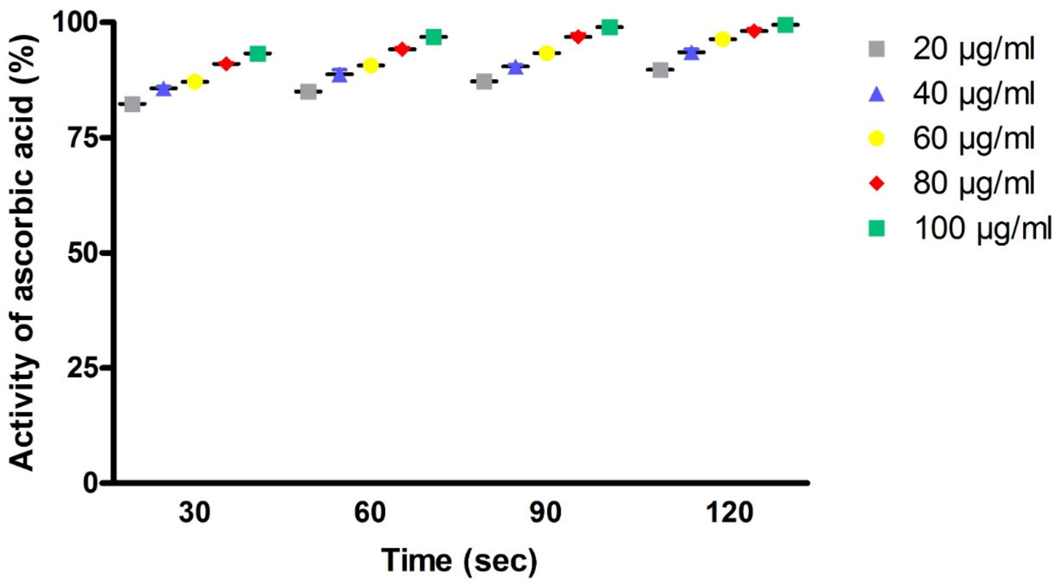

Figure S3. The activity of ascorbic acid to scavenge the free radical of DPPH at different time intervals.

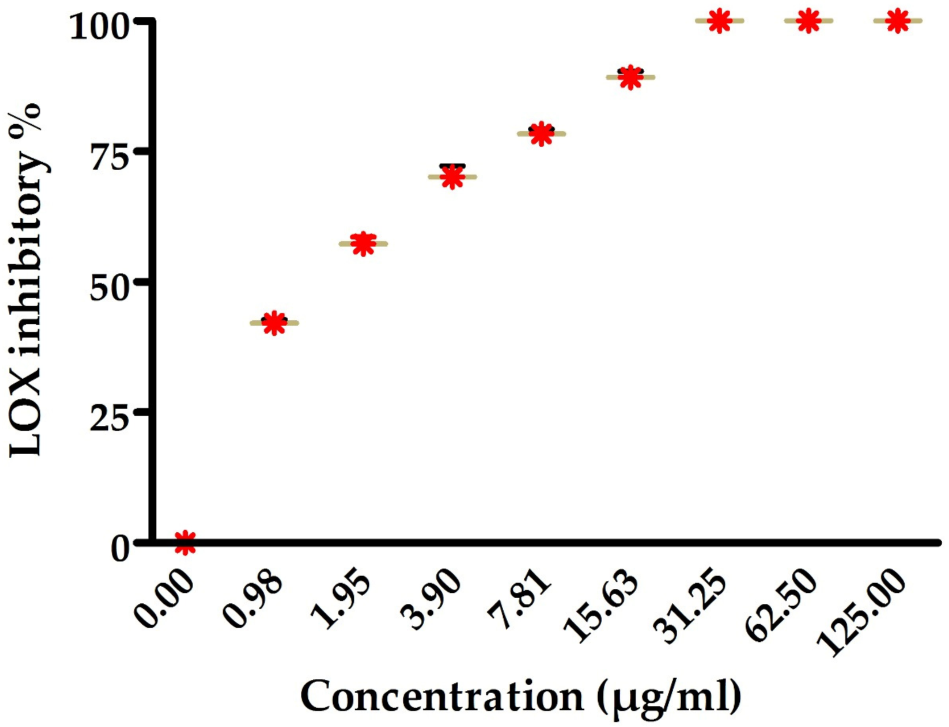

Figure S4. The inhibitory activity of ibuprofen against 5-LOX enzymatic activity.

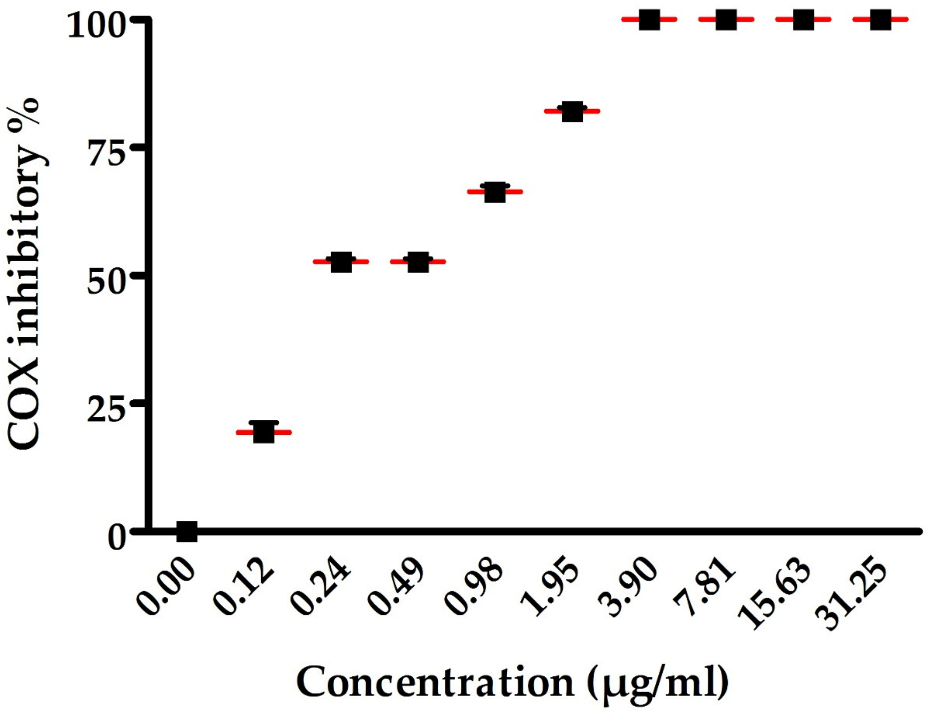

Figure S5. The inhibitory activity of celecoxib against COX-2 enzymatic activity.

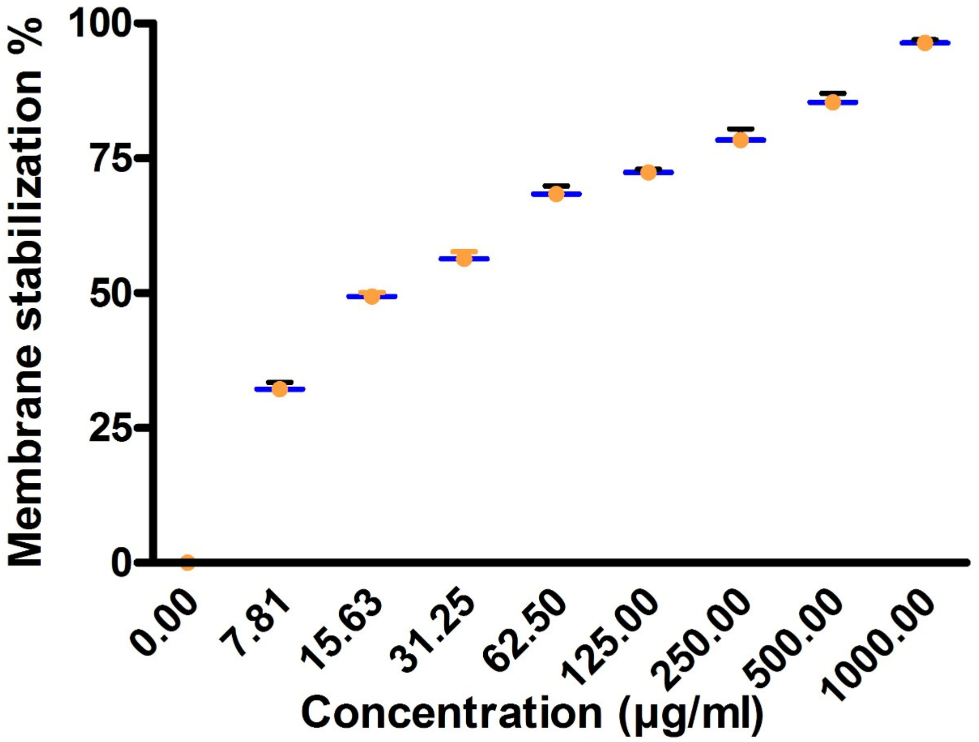

Figure S6. The inhibitory activity of indomethacin against hemolysis activity (membrane stability).

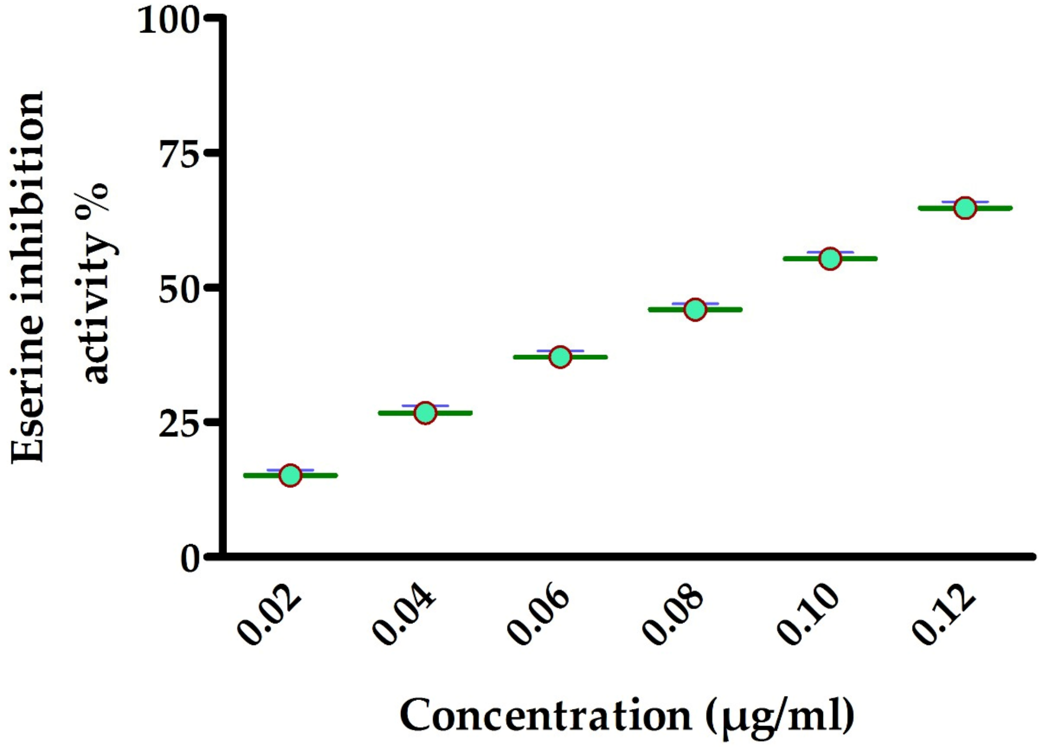

Figure S7. The inhibitory activity of Eserine against acetylcholinesterase activity.
